# Incidence Trends of Type 2 Diabetes Mellitus, Medication-Induced Diabetes, and Monogenic Diabetes in Canadian Children, Then (2006–2008) and Now (2017–2019)

**DOI:** 10.1155/2023/5511049

**Published:** 2023-11-14

**Authors:** Trisha J. Patel, Aysha Ayub, Jeffrey N. Bone, Stasia Hadjiyannakis, Mélanie Henderson, Munier A. Nour, Teresa E. Pinto, Brandy Wicklow, Jill K. Hamilton, Elizabeth A. C. Sellers, Shazhan Amed

**Affiliations:** ^1^Department of Pediatrics, University of British Columbia, 4480 Oak Street, Vancouver, BC, Canada V6H 3V4; ^2^BC Children's Hospital Research Institute, University of British Columbia, 938 West 28th Avenue, Vancouver, BC, Canada V5Z 4H4; ^3^Division of Endocrinology and Metabolism, Children's Hospital of Eastern Ontario and University of Ottawa, 401 Smyth Road, Ottawa, ON, Canada K1H 8L1; ^4^Faculty of Medicine, Université de Montréal, 3175 Côte-Sainte-Catherine, Montréal, QC, Canada H3T 1C5; ^5^Department of Pediatrics, University of Saskatchewan, 105 Administration Place, Saskatoon, SK, Canada S7N 5A2; ^6^Dalhousie University and IWK Health, 6299 South Street, Halifax, NS, Canada B3H 4R2; ^7^Department of Paediatrics and Child Health and Children's Hospital Research Institute of Manitoba, University of Manitoba, 715 McDermot Avenue, Winnipeg, MB, Canada R3E 3P4; ^8^Department of Paediatrics Hospital for Sick Children, University of Toronto, 555 University Avenue, Toronto, ON, Canada M5G 1X8

## Abstract

**Introduction:**

The landscape of childhood diabetes has evolved and addressing the knowledge gaps in non-Type 1 diabetes mellitus are key to accurate diagnosis.

**Objectives:**

A national surveillance study was completed between 2006 and 2008 and then repeated between 2017 and 2019 to describe Canadian incidence trends and clinical characteristics of non-Type 1 diabetes mellitus.

**Methods:**

We prospectively tracked new cases of non-Type 1 diabetes mellitus in children <18 years of age between June 1, 2017 and May 31, 2019. For each reported new case, a detailed questionnaire was completed, and cases were classified as Type 2 diabetes mellitus, medication-induced diabetes (MID), monogenic diabetes, or “indeterminate.” Minimum incidence rates and 10-year incidence trends of non-Type 1 diabetes mellitus and its subtypes were calculated.

**Results:**

441 cases of non-Type 1 diabetes mellitus were included (Type 2 diabetes mellitus = 332; MID = 52; monogenic diabetes = 30; indeterminate = 27). Compared to 10 years ago, the incidence of MID and monogenic diabetes remained stable, while Type 2 diabetes mellitus increased by 60% (*p* < 0.001) overall and by 37% (*p*=0.005) and 50% (*p*=0.001) in females and males, respectively. Type 2 diabetes mellitus incidence increased by 1.5 times in Indigenous (*p* < 0.001) and doubled in Asian (*p*=0.003) children.

**Conclusions:**

Canadian incidence rates of childhood-onset Type 2 diabetes mellitus have significantly increased. Further research, policy, and prevention efforts are needed to curb rising rates of youth onset Type 2 diabetes mellitus.

## 1. Introduction

Diabetes in children is a complex diagnosis with the emergence of diabetes subtypes including childhood-onset Type 2 diabetes mellitus, monogenic diabetes, and medication-induced diabetes (MID). Although the most common form of childhood-onset diabetes remains autoimmune-mediated Type 1 diabetes mellitus, our improved understanding of the pathophysiology of childhood-onset Type 2 diabetes mellitus and advances in molecular genetics identifying monogenic diabetes have allowed for better diagnostic delineation and resultant treatment of diabetes in children.

From 2006 to 2008, we reported the first Canadian minimum incidence of non-Type 1 diabetes mellitus and its subtypes in children <18 years of age with a reported observed minimum incidence rate of 1.54, 0.2, and 0.4 cases per 100,000 children per year for Type 2 diabetes mellitus, monogenic diabetes, and MID, respectively [1]. The mean age of onset of Type 2 diabetes mellitus was 13.7 years and Canadian First Nations children had the highest incidence of Type 2 diabetes mellitus. However, 50% of clinically diagnosed Type 2 diabetes mellitus occurred in non-Indigenous youth, with 25% characterized as White.

In countries such as the USA, United Kingdom, and Australia, an increasing incidence of childhood-onset Type 2 diabetes mellitus has been reported over the last decade [2, 3], however there is limited data reporting on the incidence trends in Canada. To our knowledge, there are no studies reporting on the incidence trends of MID and monogenic diabetes in children.

We conducted a prospective, National Surveillance Study in Canadian children aged <18 years from 2017 to 2019 using similar methodology to our previous study [1]. The aims of this study were to describe the: (1) incidence of non-Type 1 diabetes mellitus and its subtypes as well as the 10 year incidence trends, and (2) differences in the demographic and clinical features, diabetes-related complications, and treatment approaches for non-Type 1 diabetes mellitus subtypes between 2006–2008 and 2017–2019.

## 2. Materials and Methods

We used the Canadian Paediatric Surveillance Program (CPSP), a nationally recognized surveillance network comprised of >2,800 physicians across Canada with active medical licenses and certified in pediatrics or pediatric subspeciality. We conducted surveillance of new cases of non-Type 1 diabetes mellitus between June 1, 2017 and May 31, 2019.

### 2.1. Surveillance Methodology

After receiving a detailed case definition (Table S1), participating physicians were asked to report new cases of non-Type 1 diabetes mellitus in children <18 years of age or revised cases of non-Type 1 diabetes mellitus based on the clinical progression and/or results of investigations. To do this, they received a monthly electronic case reporting form from the CPSP. Physicians who reported “yes” to seeing a new case of non-Type 1 diabetes mellitus were sent a detailed questionnaire (available in English and French) using Research Electronic Data Capture (REDCap), or a hard copy was sent by mail upon request (see link provided with Table S1). The questionnaire collected information on the patient's demographics, family history of diabetes, birth history, anthropometrics, signs, and symptoms at presentation, and the presence of diabetic ketoacidosis (DKA) or hyperglycemic hyperosmolar state (HHS). Information on treatment, coexisting comorbidities (polycystic ovarian syndrome, hypertension, dyslipidemia, nonalcoholic liver disease, and albuminuria), and laboratory investigations (i.e., blood glucose, pH, bicarbonate, pancreatic autoantibodies, HbA_1c_, lipids, etc.) were also collected. Duplicate cases were removed using province/territory of residence, month/year of birth, sex, and date of diagnosis.

The availability of pancreatic antibody testing (i.e., GADA, ZnT8, IA-2 A, and IAA) varied across Canada. To address this, we offered no-cost pancreatic antibody testing. Physicians who indicated a need for pancreatic antibody testing on the detailed questionnaire were provided with consent and assent forms, lab requisition, and a Blood Collection kit. After consent/assent were obtained, deidentified blood samples were collected locally (during a routine blood draw) and sent via prepaid postage to the Barbara Davis Center for Childhood Diabetes in Colorado, USA [4–6]. Personnel at the Barbara Davis Center entered the results of testing into REDCap along with study ID, month/year of birth, and sex so that research staff at the BCCHR could enter results into the corresponding detailed questionnaire and share results with the reporting physician to guide clinical care. Samples were stored at the Barbara Davis Center for 3 months after which they were destroyed.

Completed detailed questionnaires were reviewed by three primary investigators (S.A., J.K.H., and E.A.C.S.) who independently assigned a diagnosis of Type 2 diabetes mellitus, monogenic diabetes, or MID. When the primary investigator assignments conflicted, the questionnaire was reviewed by the coinvestigators (B.W., M.H., T.E.P., S.H., and M.A.N.). If consensus was not achieved among the primary and coinvestigators, the case was labeled an “indeterminate” case of non-Type 1 diabetes mellitus. Cases classified as Type 1 diabetes mellitus or “other” conditions (i.e., necrotizing pancreatitis, lipodystrophy syndrome, cystic fibrosis related diabetes, etc.), as well as those deemed ineligible (i.e., insufficient data to confirm diabetes), were excluded.

Criteria for the definition of each subtype of non-Type 1 diabetes mellitus were based on:

(1) for Type 2 diabetes mellitus, the presence of risk factors as outlined in the Diabetes Canada 2018 Clinical Practice Guidelines [7] and Clinical And Demographic Information obtained from the detailed questionnaire demonstrating the natural course of the disease (i.e., presence of obesity and/or absence of pancreatic autoimmunity, and/or minimal or no insulin requirements); (2) for MID, a child receiving a known diabetogenic medication at the time of diabetes diagnosis (i.e., glucocorticoids, L-asparaginase, tacrolimus); of note, antipsychotic induced diabetes and Type 2 diabetes mellitus share similar molecular mechanisms, so exposure to an antipsychotic medication is considered a risk factor for Type 2 diabetes mellitus [7]; (3) for monogenic diabetes, confirmation of a mutation (glucokinase, hepatic nuclear factor HNF-1*α*, HNF-4*α*, HNF-1*β*, insulin promoter factor-1, neurogenic differentiation 1/*β*-cell E-box transactivator 2, KCNJ11, and ABCC8) or, when genetic testing was not pursued, a family history of diabetes affecting multiple generations in an autosomal dominant pattern and the absence of pancreatic autoimmunity [8].

Comorbidities, whenever possible, were confirmed with anthropometric measurements or laboratory results. DKA was diagnosed by the biochemical evidence of hyperglycemia (serum glucose > 11 mmol/L), acidosis (laboratory pH < 7.3 or serum bicarbonate < 15 mmol/L), and ketonemia. HHS was defined by serum bicarbonate of >15 mmol/L, serum glucose of >33 mmol/L, minimal/absent acidosis/ketosis, and serum osmolality of >320 mOsm/kg. Hypertension was defined as elevated systolic or diastolic blood pressure for age, sex, and height (based on the reference data from the American Academy of Pediatrics 2017 guideline) [9]. Nonalcoholic fatty liver disease, micro/macroalbuminuria, and dyslipidemia were confirmed based on an alanine transferase of three times the upper limit (i.e., >90 IU/L) or a finding of “fatty liver” on ultrasound [7] a random or first morning urine albumin to creatinine ratio of >2.5 mg/mmol [10] and elevated total cholesterol, LDL cholesterol, or triglycerides [11], respectively.

### 2.2. Statistical Methodology

An observed “minimum” incidence rate was calculated as the total number of new cases of non-Type 1 diabetes mellitus and its subtypes per year per 100,000 children aged <18 years. The denominators used for Canadian and province-specific incidence estimates were derived from Statistics Canada for years 2017–2019, averaged over the 24-month study period (statcan.gc.ca). Incidence rate ratios and 95% Wald confidence intervals were reported comparing minimum incidence rates previously reported in 2006–2008 to incidence rates from 2017 to 2019. [12]. Similar calculations were conducted for sex, age (≥10 and <10 years), and ethnicity subgroups. Ethnicities were collapsed into subgroups as follows: White, Black, Indigenous (First Nations, Inuit, and Metis), and Asian (Chinese, Japanese, Korean, Filipino, South-East Asian, and South Asian). Denominators for population estimates of children belonging to specific ethnic groups were not available from the recent Canadian Census and therefore, we used the 2001 denominators (statcan.gc.ca) as the reference population for both cohorts.

We conducted sensitivity analyses for possible underreporting of non-Type 1 diabetes mellitus. Like our previous study, we had no cases reported from the Territories. This study did not include family physicians or adult endocrinologists, however, in our previous study 98 family physicians and 49 adult endocrinologists reported 22 (7%) and 4 (1%) cases of non-Type 1 diabetes mellitus, respectively [1]. In the province of Quebec, legislated changes in Research Ethics Board (REB) requirements implemented August 1, 2018 (midway through our surveillance period) required academic children's hospitals to obtain REB approval to complete detailed questionnaires, increasing the possibility of underreporting. We conducted a quantitative bias analysis [13] under the following assumptions: (i) the reporting rate by family physicians and adult endocrinologists was the same as the first surveillance study in 2006–2008; (ii) all Canadian family physicians (*N* = 44,768) would report between 10% and 100% of the rate of family physicians from the first study, with a mean rate of 25%; (iii) an additional underreporting rate in Quebec between 10% and 30% compared to the first part of the current study when full reporting was feasible; and (iv) the minimum incidence rate of non-Type 1 diabetes mellitus in the Territories was between the lowest (Atlantic provinces) and the highest (Manitoba) minimum incidence rates. Corrected incidence rates were simulated 10,000 times under these assumptions. We summarized the results graphically and with summary statistics from the simulated distribution. Table S2 outlines the inputs and distributions used for the quantitative bias analysis.

### 2.3. Ethical Considerations

REB approval was obtained from the Public Health Agency of Canada and from the University of British Columbia, University of Manitoba, Hospital for Sick Children, and after August 2018, McGill University Health Center, University of Montreal, and CHU Saint-Justine. Moreover, Alberta Children's Hospital REB did not approve the completion of detailed questionnaires without patient consent (which was not feasible), however provided approval to share the number of new cases of non-Type 1 diabetes mellitus and its subtypes from 2017 to 2019.

## 3. Results and Discussion

For all CPSP studies, the average monthly response rate was 81% from 2017 to 2019. During the surveillance period, detailed questionnaire completion rates of 91.5% were comparable to 10 years prior [1]. A total of 636 cases were reported with an average of 47 cases per month. Pediatricians and pediatric endocrinologists reported 7.9% and 85.5% of cases, respectively, with the remainder (6.6%) reported by the other pediatric subspecialists.

Thirty-three (5.2%) cases were duplicated and 162 (25.5%) were excluded for reasons such as incomplete questionnaires (7.5%), not meeting the case definition (9.3%), suspected Type 1 diabetes mellitus (3.5%), and cases reported outside of the study period (5.2%).

A total of 441 cases of non-Type 1 diabetes mellitus were included for analysis: 332 cases of Type 2 diabetes mellitus, 52 cases of MID, and 30 cases of monogenic diabetes. Twenty-seven cases of non-Type 1 diabetes mellitus were classified as indeterminate. Four cases of monogenic diabetes and 16 cases of Type 2 diabetes mellitus were revised diagnoses after an initial diagnosis of Type 1 diabetes mellitus. Of note, an additional 56 cases of non-Type 1 diabetes mellitus (14 cases reported by Alberta Children's Hospital and 12 cases reported by CIUSSS-CHUS Hospital) were included in the observed minimum incidence rate analysis (Table 1).

### 3.1. Incidence and Demographics

Table 1 outlines the observed minimum incidence rate of Type 2 diabetes mellitus, MID, and monogenic diabetes between 2006 and 2008 (referred to as Cohort 1 herein) compared to 2017–2019 (referred to as Cohort 2 herein). Table S3 shows incidence rates by province.

In Cohort 2, the observed minimum incidence rates of Type 2 diabetes mellitus in females and males aged <18 years was 2.63 and 2.01 cases per 100,000 per year, respectively. Compared to Cohort 1, this represents an increase of 34% in females (incidence rate ratio (IRR): 1.34 (95% CI: 1.07, 1.68); *p*=0.009) and 50% in males (IRR: 1.50 (95% CI: 1.16, 1.95); *p*=0.001). In children <10 and ≥10 years of age in Cohort 2, the observed minimum incidence rates of Type 2 diabetes mellitus were 0.17 and 4.97 per 100,000 per year, respectively. This represents a decrease in the <10 year age group (IRR 0.65 (95% CI: 0.33, 1.30); *p*=0.22), although the confidence interval includes a possible increase. In the ≥10 year age group minimum incidence increased by 62% (IRR 1.62 (95% CI: 1.36, 1.93); *p* < 0.001).  Table 2 shows the 1-year trends in the observed minimum incidence of Type 2 diabetes mellitus by the ethnic group.

### 3.2. Sensitivity Analysis

The median bias-adjusted incidence of non-Type 1 diabetes mellitus was 28.8 (IQR: 13.7, 50.7) with conservative and maximum estimates of 3.71 and 101.9 cases per 100,000 per year, respectively (Table S2 and Figure S1). A similar analysis for Type 2 diabetes mellitus estimated a median bias-adjusted incidence of 21.1 (IQR: 10.1, 36.8) with conservative and maximum estimates of 2.62 and 74.0 cases per 100,000 children <18 years per year.

### 3.3. Clinical Findings and Investigations at Diagnosis

#### 3.3.1. Type 2 Diabetes Mellitus (N = 332)

Key demographic and clinical characteristics of Type 2 diabetes mellitus in both cohorts are shown in Table 3. The ethnic origin (not mutually exclusive) of children with new-onset Type 2 diabetes mellitus in Cohort 2 versus Cohort 1 was: White (15.4% vs. 25.1%), Indigenous (47.6% vs. 44.1%), Black (5.1% vs. 10.1%), Asian (14.6% vs. 10.1%), Hispanic (1.8% vs. 1.8%), Middle Eastern (2.1% vs. 0.4%), and mixed (8.4% vs. 6.2%).

Comorbidity was observed at diagnosis of Type 2 diabetes mellitus (Table 3). At least one comorbidity was present in 78.3% (260/332) of cases in Cohort 2 compared to only 37.4% (43/115) in Cohort 1. Three or more comorbidities were evident in 14.2% (47/332) of cases in Cohort 2 and this was similar to Cohort 1 (13.0%, 15/115). Dyslipidemia, hypertension, and albuminuria were reported more frequently in Cohort 2 compared to Cohort 1.

Regarding treatment, ∼15% of newly diagnosed children ((median HbA_1c_ 6.8% (51 mmol/mol); IQR 6.6%–7.3% (49–56 mmol/mol)) were treated with lifestyle counseling alone. There were 48.9% (157/321) of cases with an HbA_1c_ < 9% (<75 mmol/mol) that were treated with lifestyle counseling alone (26.8% (42/157)), lifestyle counseling combined with insulin (3.8% (6/157)), lifestyle counseling combined with an oral agent (59.2% (93/157)), or lifestyle counseling, insulin, and an oral agent (3.2% (5/157)). In contrast, among patients with an HbA_1c_ ≥ 9% (>75 mmol/mol) (164/321), fewer than five cases were treated with lifestyle counseling alone, whereas most were treated with lifestyle counseling combined with insulin (65.2% (107/164)), lifestyle counseling combined with an oral agent (46.3% (76/164)), or lifestyle counseling, insulin, and an oral agent (34.1% (56/164)).

#### 3.3.2. Medication-Induced Diabetes (Table 4; N = 52)

Children presented at a mean ± SD age of 13.47 ± 3.42 years; 40.4% (21/52) were White and 29.2% (14/48) were obese. Fifty-eight percent (30/52) were asymptomatic at diagnosis. Polyuria (31% (16/52)) and polydipsia (25% (13/52)) were the most common symptoms. The average HbA_1c_ at presentation was 7.2% (55 mmol/mol) ± 2.0% and the median HbA_1c_ was 6.7% (50 mmol/mol). Glucocorticoid therapy was reported in 96% (50/52) of children; isolated glucocorticoid treatment was reported in 60% (31/52) and glucocorticoids in combination with tacrolimus, L-asparaginase, or cyclosporine in 40.4% (21/52). Less than five children did not receive treatment for their diabetes, whereas in Cohort 1, 14% (7/52) of cases of MID did not receive treatment. Insulin therapy alone (46.2% (24/52)), and a combination of insulin and lifestyle counseling (51.9% (27/52)) were used at similar frequencies. Lifestyle counseling alone was used in fewer than five cases.

#### 3.3.3. Monogenic Diabetes (Table 4; N = 30)

Children presented at a mean ± SD age of 10.70 ± 4.28 years, and 43.3% (13/30) were White. The majority were asymptomatic (67% (20/30)). In those with symptoms, polyuria (20% (6/30)) and polydipsia (20% (6/30)) were most common. Acanthosis nigricans was reported in fewer than five children. The mean BMI *z*-score at diagnosis was 0.51 ± 1.33. Twenty-one percent (6/29) were overweight, and 21% (6/29) were obese at presentation. The mean HbA_1c_ ± SD at presentation was 7.4% (57 mmol/mol) ± 1.8% and the median HbA_1c_ was 6.7% (50 mmol/mol). Of those tested for antibodies, GADA (21/30), IA-2A (10/30), and IAA (8/30) were negative in all children. Results of the genetic testing were available in 29 patients; most cases (19/29) had glucokinase mutations with a minority of mutations in the HNF-1 *α*, HNF-1 *β*, insulin promoter factor-1 (INS), or KLF11 genes. There were no cases of neonatal diabetes. Treatment was not initiated in 36.7% (11/30) of children. Of those treated, regimens primarily included insulin alone (13.3% (4/30)) or lifestyle counseling alone (26.7% (8/30)). Fewer than five cases were managed with a combination of insulin and lifestyle counseling. All children with HNF-1 *α* mutations were treated with an oral hypoglycemic agent alone. Most children with a new diagnosis of monogenic diabetes did not have comorbidities (70% (21/30)).

## 4. Conclusions

Over the last decade, the observed minimum incidence of non-Type 1 diabetes mellitus in children <18 years of age increased in Canada, largely due to increasing rates of Type 2 diabetes mellitus in children ≥10 years of age. To our knowledge, our study is the first to report stable observed minimum incidence rates of MID and monogenic diabetes in children. Incidence of Type 2 diabetes mellitus significantly increased in both females and males with a higher rate of increase in males versus females (50% vs. 37%). Also, incidence significantly increased in children of Asian and Indigenous background.

The increasing incidence of Type 2 diabetes mellitus in Canadian children is higher than that reported in the UK, where, using a similar surveillance methodology, the incidence of Type 2 diabetes mellitus in children <17 years of age increased from 0.53 cases per 100,000 (95% CI: 0.41–0.68) in 2004–2005 to 0.72 cases per 100,000 (95% CI: 0.58–0.88) [14] in 2015–2016. Other countries around the world also report an increasing incidence of childhood-onset Type 2 diabetes mellitus. The US SEARCH study reported an annual percent change in the incidence of Type 2 diabetes mellitus in children aged 10–19 years of age of 4.8% from 2002 to 2015 (from 9.0 cases per 100,000 youths per year in 2002–2003 to 12.5 cases per 100,000 youths per year in 2011–2012), after adjustment for age, sex, and race or ethnic group. [3, 15]. The higher incidence of Type 2 diabetes mellitus reported in the US SEARCH study [3, 15] may be due in part to methodological differences for case ascertainment, an overrepresentation of ethnic minorities, a higher prevalence of childhood obesity (13% in 2009–2013 in Canada vs. 17.5% in 2009–2012) [16] which is a known risk factor for Type 2 diabetes mellitus, and the variable impact of social determinants of health. In Western Australia, from 2000 to 2019, the incidence of Type 2 diabetes mellitus in children <16 years of age increased on average by 5.2% (95% CI: 2.8–7.8) per year with an overall mean incidence of 2.3 per 100,000 youths per year (95% CI: 2.1–2.7) [2].

We found that the rate of increase in minimum incidence over the two time periods was higher in males compared to females. However, the overall proportion of female youth affected continues to remain higher compared to males, consistent with other published data and our first surveillance study. The US SEARCH study [3, 15] reported the annual increase in incidence of childhood-onset Type 2 diabetes mellitus in males was lower than in females (from 2002–2003 to 2011–2012). Aside from the different time periods of data collection, we were unable to identify any plausible explanations from published literature on why our Canadian data are different from the United States. Notably, prevalence of obesity in Canadian male youth is higher compared to the female youth [17]. More research is needed to better understand the sociobiological differences in males and females and their impact on risk for Type 2 diabetes mellitus.

We found the highest incidence of childhood-onset Type 2 diabetes mellitus in Canadian Indigenous (predominantly First Nations) children. In the UK, Asian children had the highest (2.92 cases per 100,000 per year) while White children had the lowest incidence (0.78 cases per 100,000 per year) of Type 2 diabetes mellitus [14]. In our study, the incidence of Type 2 diabetes mellitus remained relatively stable in White and Black children but increased by 1.5 times in Indigenous and doubled in Asian children. In the United States, between 2002–2010 and 2011–2015, the steepest annual percent change in childhood-onset Type 2 diabetes mellitus incidence was in Asians and Pacific Islanders (7.7% per year), followed by Hispanics (6.5% per year), Blacks (6.0% per year), and American Indians (3.7% per year) [15].

The prevalence of overweight and obesity in children aged 12–17 years has remained relatively stable from 2015 to 2020 [18] yet, over the last decade, rates of Type 2 diabetes mellitus have continued to rise in children ≥10 years of age. Compared to 10 years ago, we report no change in the number of cases of newly diagnosed Type 2 diabetes mellitus in children <10 years of age. In children with MID and monogenic diabetes, rates of obesity are higher compared to the unaffected Canadian youth where the median BMI *z*-score was 0.27 (−0.39–1.23) in 2012–2013 for age 3–19 years [17]. This trend was observed in Canadian children with acute lymphoblastic leukemia (ALL) and MID < 18 years of age where 50% of cases had a BMI greater than the 95th percentile, compared to no cases in matched controls with ALL (*p*=0.005) [19]. The US SEARCH study also described obesity in youth with genetically confirmed and unconfirmed maturity-onset diabetes of the young (MODY) [20].

Compared to Canadian children diagnosed with Type 2 diabetes mellitus in 2006–2008, a similar proportion of cases in 2017–2019 had obesity (87.9% vs. 86.3%) but had a higher BMI median *z*-score (2.93 vs. 2.84). The degree of obesity at diagnosis of childhood-onset Type 2 diabetes mellitus varies across ethnic groups. We previously reported that First Nations children with a new diagnosis of Type 2 diabetes mellitus had significantly lower BMI *z*-scores compared to the White children and those from other high risk ethnic groups [21]. In this study, the mean BMI *z*-score for White children was higher compared to Asian children (2.47 vs. 1.85; mean difference 0.62 (95% CI: 0.09, 1.14)). The UK similarly showed that Asian children with Type 2 diabetes mellitus had a statistically lower mean BMI *z*-score (−0.53 (−0.81, −0.24); *p*=0.01) compared to the White children [14]. This supports the idea that children belonging to some ethnic/racial minority groups have an increased risk for developing Type 2 diabetes mellitus at a lower BMI threshold. Indeed, transcription factor variants have been identified that increase the risk of Type 2 diabetes mellitus in African American [22] and First Nations children [23, 24] however, social determinants of health such as poverty, food insecurity, and obesogenic environments, as well as in utero exposure to diabetes, are key contributors to the intergenerational Type 2 diabetes mellitus [25].

Like other studies [26], we found treatment of Type 2 diabetes mellitus in Canadian children consists predominantly of lifestyle counseling, metformin monotherapy or in combination with insulin. In Cohort 2, ∼15% of cases received lifestyle counseling alone, with no initiation of an insulin sensitizer at diagnosis despite ADA 2023 [27] and ISPAD 2022 [28] guidelines recommending metformin as an initial treatment in metabolically stable youth. This finding highlights a knowledge gap and the need for increased awareness and education among pediatric health-care providers. Our advanced understanding of the pathophysiology of Type 2 diabetes mellitus in children suggests that these treatment approaches may not be effective. The TODAY trial showed that metformin alone or combined with insulin does not curb the rapid decline in *β*-cell function (reported to be twice that of adults) in the subpopulations of children [29, 30]. Moreover, the RISE Study showed that insulin and metformin are ineffective in preventing *β*-cell decline in youth with both prediabetes and recently diagnosed Type 2 diabetes mellitus [31–33]. Until recently, treatment of childhood-onset Type 2 diabetes mellitus has been limited to metformin and insulin, however, new drugs approved for use in pediatric Type 2 diabetes mellitus such as glucagon-like peptide (GLP-1) agonists show promise [34].

Compared to 10 years ago, there were minimal differences in the characteristics of children with MID and monogenic diabetes. Interestingly, fewer children diagnosed with MID in 2017–2019 had obesity (29% vs. 52%) and very few were left untreated (2% vs. 7%). This supports evidence that more aggressive treatment of hyperglycemia in children with underlying chronic disease may improve outcomes [35]. Ten years ago, half of the patients with a clinical diagnosis of monogenic diabetes had genetic testing, while in this study, all but one patient had a confirmed genetic diagnosis, substantiating improved access to molecular genetic testing. In contrast to prevalence, the minimum incidence rate for monogenic diabetes, nationally or globally, has not been reported. Subgroup epidemiological data have been published. The minimum incidence of MODY in patients aged 1–18 years was 1.2% in a Swedish cohort [36]. The minimum incidence of neonatal diabetes was estimated at 1 : 90,000 live births in Italy [37] and 1 : 89,000 live births in Germany [38]. Although direct comparison cannot be made between studies, we postulate the minimum incidence rate may be lower in our study because cases of monogenic diabetes may have been misdiagnosed as Type 1 diabetes mellitus. Such cases were not analyzed and access to molecular genetic testing may have been limited.

This study has limitations that are important to consider. Our surveillance methodology generated a minimum incidence rate because patients with non-Type 1 diabetes mellitus seen by nonparticipating clinicians (i.e., adult endocrinologists, family physicians, and nurse practitioners) were not captured. Assessment of completeness of ascertainment was not possible (i.e., using independent sources of data such as prescription or hospitalization data) because many children with Type 2 diabetes mellitus are not receiving medications and hospitalizations are not frequent.

Given the complexity of the diagnosis [39], all cases of non-Type 1 diabetes mellitus may not have been recognized by reporting physicians and incomplete detailed questionnaires hindered confirmation and classification of the diabetes subtype. We experienced variations in provincial REB requirements that may have led to underreporting. Moreover, under-reporting in Saskatchewan in our previous study with marked improvement to report in this study resulted in an inaccurate 10-year incidence trend estimate for this province. Despite missing data, the survey response rate was high with relatively wide geographic representation.

More recent population estimates stratified by ethnicity were not available from the Statistics Canada and therefore, we used data from 2001 to calculate incidence rates of Type 2 diabetes mellitus by an ethnic group, which may not have been representative of the current population demographics. Moreover, ethnicity was physician-reported. Although offered at no cost to reporting physicians, many cases had incomplete or no pancreatic antibody testing. Furthermore, a very small subset of patients classified as indeterminate had low levels of pancreatic autoimmunity and might have evolved to Type 1 diabetes mellitus. Cases of monogenic diabetes might have been misclassified as Type 2 diabetes mellitus if genetic testing was not completed, as was shown in the TODAY study where 4.5% of youth with a clinical diagnosis of Type 2 diabetes mellitus were found to have a pathogenic variant known or likely to cause monogenic diabetes [40]. In last, comorbidities such as microalbuminuria and dyslipidemia may have been over-misdiagnosed based on single laboratory samples or samples collected during metabolic decompensation. A diagnosis of hypertension was based on a single blood pressure measurement as opposed to three consecutive blood pressure measurements and/or ambulatory blood pressure monitoring. This may explain the higher proportion of youth with at least one comorbidity at diagnosis in our study, compared to what has been reported in the US SEARCH study [41] and the TODAY study [42]. Further, there is no consensus on the gold standard for diagnosing NAFLD so the ALT threshold value and positive ultrasound findings lack high sensitivity and specificity for diagnosis.

Study strengths include that it is population-based, involved providers (including subspecialists) from across Canada, and data available from 10 years ago allowed for the description of incidence trends, as well as a quantitative bias sensitivity analysis. Some Canadian provinces have reported on the incidence and prevalence trends of childhood-onset Type 2 diabetes mellitus [43] however these studies are province-specific and used administrative health data which are not comparable to the surveillance data used in this study. We are confident that most cases of non-Type 1 diabetes mellitus were captured during the surveillance period as the model of care for pediatric chronic disease in most provinces, particularly for rare diseases such as monogenic diabetes or Type 2 diabetes mellitus, is referral to pediatric practitioners. In last, we gathered important data on differences in the treatment of non-Type 1 diabetes mellitus and its subtypes today versus 10 years ago.

This prospective national surveillance study expands our understanding of the spectrum of non-Type 1 diabetes mellitus. In Canada, like the rest of the world, Type 2 diabetes mellitus in children is increasing, putting them at 2–3 times higher risk for mortality compared to the general population [44]. Children ≥10 years of age, female youth, and those who are Asian and Indigenous may benefit from codesigned, multicomponent, culturally, and age-appropriate prevention interventions that truly meet the unique needs of these populations. Continued surveillance of Type 2 diabetes mellitus in children will be critical to track the success of prevention interventions, as well as characterize the evolution in treatment strategies over time as innovative, multicentre, and multinational clinical trials [45] identify more effective treatment approaches.

## Figures and Tables

**Table 1:** Comparison of observed minimum incidence rates in Cohorts 1 and 2.

	Cohort 1 2006–2008	Cohort 2 2017–2019	Cohort 2 vs. Cohort 1	
Type	Incidence per 100,000 per year	Incidence per 100,000 per year	Incidence rate ratio (95% CI)	*p*-Value
Non-Type 1 diabetes	2.34	3.40	1.45 (1.26, 1.66)	<0.001
Type 2 diabetes mellitus	1.54	2.47	1.60 (1.36, 1.89)	<0.001
Monogenic diabetes	0.21	0.23	1.09 (0.67, 1.78)	0.73
Medication-induced diabetes	0.38	0.44	1.17 (0.82, 1.68)	0.38
Indeterminate	0.22	0.24	1.16 (0.71, 1.88)	0.55

*Note*: For Cohort 1, population estimates came from 2006 Canadian Census—Statistics Canada. For Cohort 2, the reference population is the average population during those years of Canadian children aged 0–17 years.

**Table 2:** Comparison of observed minimum incidence rates of Type 2 diabetes mellitus in Cohorts 1 and 2 by ethnicity.

	Cohort 1	Cohort 2	Cohort 2 vs. Cohort 1	
	2006–2008	2017–2019		
	Incidence per 100,000 per year ^*∗*^	Incidence per 100,000 per year ^*∗*^	Incidence rate ratio (95% CI)	*p*-Value
White	0.54	0.49	0.89 (0.61, 1.31)	0.56
Indigenous^a^	23.4	36.6	1.56 (1.22, 2.00)	<0.001
Black	7.8	5.7	0.74 (0.39, 1.38)	0.34
Asian^b^	1.9	4.0	2.08 (1.27, 3.43)	0.003

*Note*:  ^*∗*^Population estimate data from 2001 Canadian Census–Statistics Canada. ^a^Indigneous ethnicity includes: First Nations, Metis, and Inuit. ^b^Asian ethnicity includes: Cohort 1-Chinese, Japanese, Filipino, Vietnamese, Indian, Pakistani; Cohort 2-Chinese, Japanese, Korean, Filipino, South East Asian, and South Asian.

**Table 3:** Comparison of clinical characteristics of Type 2 diabetes mellitus at presentation in Cohorts 1 and 2.

	Cohort 1 2006–2008 (*N* = 227)	Cohort 2 2017–2019 (*N* = 332)
Age		
Median years (IQR)	14.0 (12.3, 15.7)	14.1 (12.5, 15.7)
<10 years (%)	18 (7.9)	14 (4.2)
Sex		
Female (%)	132 (58)	185 (55.7)
Family history		
Affected family member^a^ (%)	185 (91)	294 (88.6)
In-utero diabetes exposure^b^ (%)	81 (35.7)	147 (44.3)
Hemoglobin A1c		
Mean (SD)	9.6 (3.0)	9.6 (2.7)
Median (IQR)	8.7 (5.4, 18.2)	9.1 (5.3, 11.7)
BMI		
Median (IQR)	31.0 (27.1, 35.8)	33.3 (28.3, 38.1)
Median *z*-score (IQR)	2.84 (2.30, 3.57)	2.93 (2.29, 3.84)
BMI group		
Normal weight (%)	10 (4.4)	10 (3.0)
Overweight ^*∗*^ (%)	10 (4.4)	17 (5.1)
Obese ^*∗*^ (%)	196 (86.3)	292 (87.9)
Missing (%)	11 (4.9)	13 (3.9)
Asymptomatic at diagnosis (%)	85 (37.4)	146 (44.0)
Diabetic ketoacidosis (%)	22 (9.7)	23 (6.9)
Comorbidity		
Polycystic ovarian syndrome (%)	16/132 (12.1)	27/305 (8.9)
Dyslipidemia (%)	78/174 (44.8)	183/279 (65.6)
Hypertension (%)	58/205 (28.3)	142/302 (47.0)
Nonalcoholic fatty liver disease (%)	39/176 (22.2)	74/282 (26.2)
Albuminuria (%)	21/148 (14.2)	59/228 (25.9)

*Note*: ^a^First- or second-degree relative. ^b^Gestational diabetes mellitus, maternal Type 1 diabetes mellitus, or maternal Type 2 diabetes mellitus.  ^*∗*^Greater than 85th (overweight) and 95th (obesity) percentile, respectively, based on WHO Growth Charts for Canada 2014.

**Table 4:** Comparison of clinical characteristics of monogenic diabetes and medication-induced diabetes at presentation in Cohorts 1 and 2.

	Monogenic diabetes		Medication-induced diabetes	
	Cohort 1 2006–2008 (*N* = 30)	Cohort 2 2017–2019 (*N* = 30)	Cohort 1 2006–2008 (*N* = 55)	Cohort 2 2017–2019 (*N* = 52)
Median age (IQR)	11.75 (0, 17.75)	10.85 (8.01, 13.11)	14.58 (3.67, 17.83)]	13.95 (11.68, 16.35)
Female (%)	17 (56.7)	13 (43.0)	30 (54.5%)	22 (42.0)
Hemoglobin A1c				
Mean (SD)	7.4 (2.4)	7.4 (1.8)	6.6 (1.9)	7.2 (2.0)
Median (IQR)	6.7 (4.4, 15)	6.7 (5.9, 13.0)	5.9 (4.8, 12.7)	6.7 (4.9, 14.0)
Median BMI (IQR)	18.1 (8.87, 27.4)	19.3 (15.5, 23.4)	22.7 (11.1, 39.8)	20.8 (18.2, 24.3)
Median BMI *z*-score	0.30 (−1.42, 0.96)	0.65 (−0.31, 1.40)	1.05 (0.02, 2.64)	0.62 (−0.35, 1.83)

## Data Availability

The datasets generated during and/or analyzed in the current study are available from the corresponding author upon reasonable request.

## Supplementary Materials

Supplementary Materials Table S1: case definition of non-Type 1 diabetes provided to reporting physicians. Table S2: inputs and distributions for the quantitative bias analyses (sensitivity analysis). Table S3: minimum incidence rates (per 100,000 children per year) for Type 2 diabetes, monogenic diabetes, and medication-induced diabetes for Cohorts 1 and 2 stratified by Canadian province. Figure S1: results of the sensitivity analysis in the form of a histogram showing the bias adjusted incidence rate per 100,000 per year of non-Type 1 diabetes.

## References

[1] Amed S., Dean H. J., Panagiotopoulos C. (2010). Type 2 diabetes, medication-induced diabetes, and monogenic diabetes in Canadian children: a prospective national surveillance study. *Diabetes Care*.

[2] Haynes A., Curran J. A., Davis E. A. (2021). Two decades of increasing incidence of childhood-onset Type 2 diabetes in Western Australia (2000–2019). *Medical Journal of Australia*.

[3] Mayer-Davis E. J., Lawrence J. M., Dabelea D. (2017). Incidence trends of Type 1 and Type 2 diabetes among youths, 2002–2012. *New England Journal of Medicine*.

[4] Yu L., Robles D. T., Abiru N. Early expression of antiinsulin autoantibodies of humans and the NOD mouse: evidence for early determination of subsequent diabetes.

[5] Yu L., Boulware D. C., Beam C. A. (2012). Zinc transporter-8 autoantibodies improve prediction of Type 1 diabetes in relatives positive for the standard biochemical autoantibodies. *Diabetes Care*.

[6] Bonifacio E., Yu L., Williams A. K. (2010). Harmonization of glutamic acid decarboxylase and islet antigen-2 autoantibody assays for National Institute of Diabetes and digestive and kidney diseases consortia. *The Journal of Clinical Endocrinology & Metabolism*.

[7] Diabetes Canada Clinical Practice Guidelines Expert Committee, Panagiotopoulos C., Hadjiyannakis S., Henderson M. (2018). Type 2 diabetes in children and adolescents. *Canadian Journal of Diabetes*.

[8] Amed S., Oram R. (2016). Maturity-onset diabetes of the young (MODY): making the right diagnosis to optimize treatment. *Canadian Journal of Diabetes*.

[9] Flynn J. T., Kaelber D. C., Baker-Smith C. M. (2017). Clinical practice guideline for screening and management of high blood pressure in children and adolescents. *Pediatrics*.

[10] Dart A. B., Sellers E. A., Martens P. J., Rigatto C., Brownell M. D., Dean H. J. (2012). High burden of kidney disease in youth-onset Type 2 diabetes. *Diabetes Care*.

[11] Expert Panel on Integrated Guidelines for Cardiovascular Health and Risk Reduction in Children and Adolescents (2011). Expert Panel On Integrated Guidelines for Cardiovascular Health and Risk Reduction in Children and Adolescents: Summary Report. *Pediatrics*.

[12] Rothman K. J. (2012). *Epidemiology: An Introduction*.

[13] Lash T. L., Fox M. P., Fink A. K. (2009). *Applying Quantitative Bias Analysis to Epidemiologic Data*.

[14] Candler T. P., Mahmoud O., Lynn R. M., Majbar A. A., Barrett T. G., Shield J. P. H. (2018). Continuing rise of Type 2 diabetes incidence in children and young people in the UK. *Diabetic Medicine*.

[15] Divers J., Mayer-Davis E. J., Lawrence J. M. (2020). Trends in incidence of Type 1 and Type 2 diabetes among youths—selected counties and Indian reservations, United States, 2002–2015. *MMWR. Morbidity and Mortality Weekly Report*.

[16] Centers for Disease Control and Prevention (CDC). National Center for Health Statistics (NCHS) (2009–2012). National health and nutrition examination survey data. *Centers for Disease Control and Prevention*.

[17] Rodd C., Sharma A. K. (2016). Recent trends in the prevalence of overweight and obesity among Canadian children. *Canadian Medical Association Journal*.

[18] Statistics Canada (2022). Table 13-10-0096-21 body mass index, overweight or obese, self reported, youth (12 to 17 years old). *Health characteristics, Annual Estimates*.

[19] Ho J., Sellers E., Dean H. (2010). Prevalence and associated risk factors for secondary diabetes in Canadian children followed in pediatric tertiary care centres. *Canadian Journal of Diabetes*.

[20] Pihoker C., Gilliam L. K., Ellard S. (2013). Prevalence, characteristics and clinical diagnosis of maturity onset diabetes of the young due to mutations in HNF1A, HNF4A, and glucokinase: results from the SEARCH for diabetes in youth. *The Journal of Clinical Endocrinology & Metabolism*.

[21] Amed S., Hamilton J. K., Sellers E. A. C. (2012). Differing clinical features in aboriginal vs. non-aboriginal children presenting with Type 2 diabetes. *Pediatric Diabetes*.

[22] Dabelea D., Dolan L. M., D’Agostino R. (2011). Association testing of TCF7L2 polymorphisms with Type 2 diabetes in multi-ethnic youth. *Diabetologia*.

[23] Hegele R. A. (1999). The hepatic nuclear factor-1 G319S variant Is associated with early-onset Type 2 diabetes in Canadian Oji-Cree. *Journal of Clinical Endocrinology & Metabolism*.

[24] Sellers E. A. C., Triggs-Raine B., Rockman-Greenberg C., Dean H. J. (2002). The prevalence of the HNF-1*α* G319S mutation in Canadian aboriginal youth with Type 2 diabetes. *Diabetes Care*.

[25] Prinjha S., Wicklow B., Nakhla M., Banerjee A. T. (2022). Toward the goal of understanding and tackling the social determinants of diabetes. *Canadian Journal of Diabetes*.

[26] Badaru A., Klingensmith G. J., Dabelea D. (2014). Correlates of treatment patterns among youth with Type 2 diabetes. *Diabetes Care*.

[27] ElSayed N. A., Aleppo G., Aroda V. R. (2023). 14. Children and adolescents: standards of care in diabetes—2023. *Diabetes Care*.

[28] Shah A. S., Zeitler P. S., Wong J. (2022). ISPAD clinical practice consensus guidelines 2022: Type 2 diabetes in children and adolescents. *Pediatric Diabetes*.

[29] TODAY Study Group, Zeitler P., Hirst K. (2012). A clinical trial to maintain glycemic control in youth with Type 2 diabetes. *New England Journal of Medicine*.

[30] TODAY Study Group (2013). Effects of metformin, metformin plus rosiglitazone, and metformin plus lifestyle on insulin sensitivity and *β*-cell function in TODAY. *Diabetes Care*.

[31] Zeitler P., Hirst K., Copeland K. C. (2015). HbA1c after a short period of monotherapy with metformin identifies durable glycemic control among adolescents with Type 2 diabetes. *Diabetes Care*.

[32] Kahn S. E., Haffner S. M., Heise M. A. (2006). Glycemic durability of rosiglitazone, metformin, or glyburide monotherapy. *New England Journal of Medicine*.

[33] The RISE Consortium (2018). Impact of insulin and metformin versus metformin alone on *β*-cell function in youth with impaired glucose tolerance or recently diagnosed Type 2 diabetes. *Diabetes Care*.

[34] Tamborlane W. V., Barrientos-Pérez M., Fainberg U. (2019). Liraglutide in children and adolescents with Type 2 diabetes. *New England Journal of Medicine*.

[35] Acosta-Gualandri A., Blydt-Hansen T., Islam N., Amed S. (2021). Risk factors for developing posttransplant diabetes after pediatric kidney transplant in a canadian tertiary care children’s hospital between 1995 and 2016. *Canadian Journal of Diabetes*.

[36] Carlsson A., Shepherd M., Ellard S. (2020). Absence of islet autoantibodies and modestly raised glucose values at diabetes diagnosis should lead to testing for MODY: lessons from a 5-year pediatric Swedish National Cohort Study. *Diabetes Care*.

[37] (2012). Minimal incidence of neonatal/infancy onset diabetes in Italy is 1 : 90,000 live births. *Acta Diabetologica*.

[38] Grulich-Henn J., Wagner V., Thon A. (2010). Entities and frequency of neonatal diabetes: data from the diabetes documentation and quality management system (DPV). *Diabetic Medicine*.

[39] Amed S., Pozzilli P. (2016). Diagnosis of diabetes type in children and young people: challenges and recommendations. *The Lancet Diabetes & Endocrinology*.

[40] Todd J. N., Srinivasan S., Pollin T. I. (2018). Advances in the genetics of youth-onset Type 2 diabetes. *Current Diabetes Reports*.

[41] Koebnick C., Imperatore G., Jensen E. T. (2020). Progression to hypertension in youth and young adults with Type 1 or Type 2 diabetes: The SEARCH for Diabetes in Youth Study. *The Journal of Clinical Hypertension*.

[42] TODAY Study Group (2013). Rapid rise in hypertension and nephropathy in youth with Type 2 diabetes. *Diabetes Care*.

[43] Amed S., Islam N., Sutherland J., Reimer K. (2018). Incidence and prevalence trends of youth-onset Type 2 diabetes in a cohort of Canadian youth: 2002–2013. *Pediatric Diabetes*.

[44] Constantino M. I., Molyneaux L., Limacher-Gisler F. (2013). Long-term complications and mortality in young-onset diabetes: Type 2 diabetes is more hazardous and lethal than Type 1 diabetes. *Diabetes Care*.

[45] Pyle L., Kelsey M. M. (2021). Youth-onset Type 2 diabetes: translating epidemiology into clinical trials. *Diabetologia*.

