# Supplementary material for: Incidence Trends of Type 2 Diabetes Mellitus, Medication-Induced Diabetes, and Monogenic Diabetes in Canadian Children, Then (2006–2008) and Now (2017–2019)
Source: Pediatr Diabetes. 2023 Nov 14;2023:5511049. doi: 10.1155/2023/5511049 (PMC12017104; doi:10.1155/2023/5511049)

**Supplementary Table 1.** Case Definition of Non-Type 1 Diabetes Mellitus (https://cpsp.cps.ca/uploads/studies/Non-type-1-diabetes-questionnaire-.pdf)


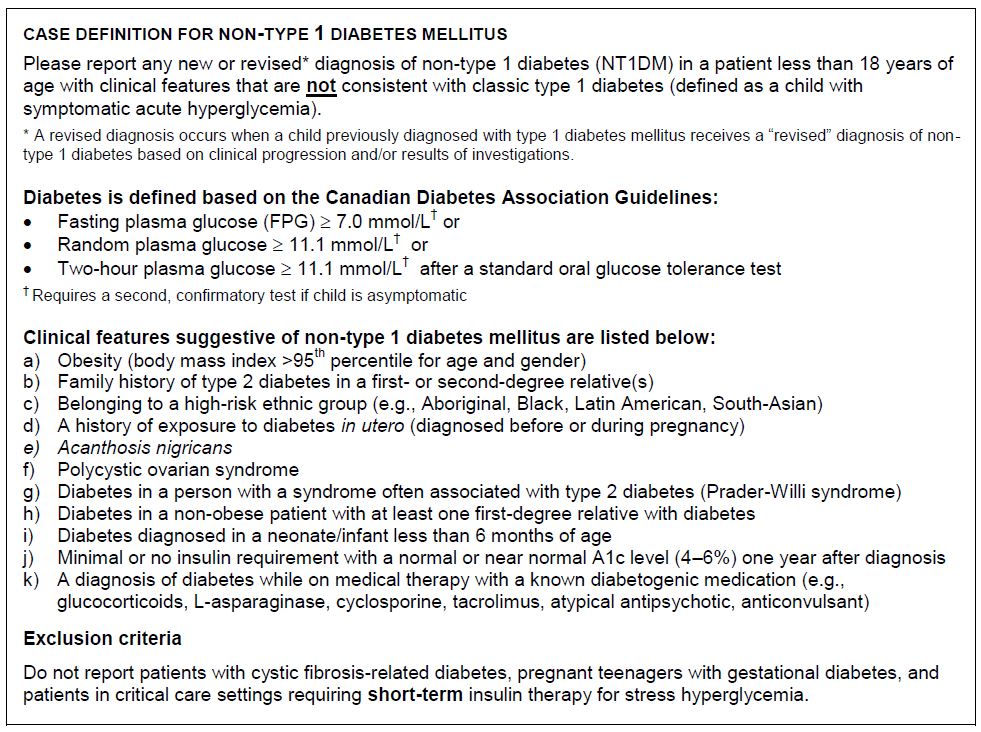


**Supplementary Table 2.** Inputs and Distributions for Quantitative Bias Analyses

| **Parameters** | **Source** | **Assumed distribution** | **Parameters** | **Estimated additional cases (repeated 10,000 times)** |
| --- | --- | --- | --- | --- |
| Family doctors captured in first study but not second | First surveillance study reported 22/345 = 0.0637 proportion of cases from 98 sampled family doctors | Normal | Mean = 0.0637    Variance = 0.00017 | 485 (total cases) X output from distribution |
| All Canadian family doctors (N = 44768) | Distribution set to assume that other family doctors report at between 0 and 100% of the rate of those above, with a mean rate of 30% | Beta | Mean =  0.308    Variance = 0.059 | 44768 (total doctors) X 0.315* X output from distribution    *Estimated as the number of cases per family physician in study 1 times the increase in cases from study 1 to 2 |
| Adult endocrinologist | First surveillance study reported 4/345 = 0.011 proportion of cases from adult endocrinologists | Normal | Mean = 0.011    Variance = 3.3 x10^-5^ | 485 (total cases) X output from distribution |
| Quebec underreporting | Based on expert input. | Uniform | Minimum = 10% underreporting    Maximum = 30% underreporting | 45* X output from distribution    *number of total cases in Quebec |
| Territory data (population = 32966) | Based on lowest (Atlantic) and highest (Manitoba) incidence rates observed in study | Uniform | Minimum = 1.43 per 100,000    Maximum = 15.3 per 100,000 | 100000 X output from distribution/32966 |

Final outputs were calculated per 100,000 per year for total Canadian population

**Supplementary Table 3**. Provincial minimum incidence rates (per 100,000 children per year) of type 2 diabetes mellitus, monogenic diabetes, and medication-induced diabetes in Canadian children aged <18 years for cohort 1 (2006**–**2008) and cohort 2 (2017**–**2019)

|  | **Type 2 Diabetes Mellitus** | | **Monogenic Diabetes** | | **Medication-Induced Diabetes** | |  |
| --- | --- | --- | --- | --- | --- | --- | --- |
|  | **Cohort 1**  **2006–2008**  **(N=227)** | **Cohort 2**  **2017–2019**  **(N=354)** | **Cohort 1**  **2006–2008**  **(N=30)** | **Cohort 2**  **2017–2019**  **(N=33)** | **Cohort 1**  **2006–2008**  **(N=55)** | **Cohort 2**  **2017–2019**  **(N=64)** | |
| **Canada** | 1.54 | 2.47 | 0.21 | 0.23 | 0.38 | 0.44 | |
| **Alberta^1^** | 0.7 | 2.30 | 0.2 | 0.42 | 0.15 | 0.57 | |
| **Atlantic** | 0.7 | 1.20 | 0.05 | 0.12 | 0.2 | 0.24 | |
| **British Columbia** | 1.2 | 1.38 | 0.25 | 0.23 | 0.2 | 0.81 | |
| **Manitoba** | 12.45 | 14.40 | 0.55 | 0.00 | 0.9 | 0.98 | |
| **Ontario** | 1.7 | 1.84 | 0.2 | 0.18 | 0.6 | 0.44 | |
| **Quebec^2^** | 0.55 | 0.99 | 0.3 | 0.16 | 0.2 | 0.16 | |
| **Saskatchewan** | 0.4**^3^** | 10.40 | 0 | 0.93 | 0 | 0.19 | |
| **Territories** | 0 | - | 0 | 0 | - | 0 | |

^1^Cases from Alberta Children’s Hospital (N=44) were included in case counts but not in further analyses as local ethical approval was not granted. ^2^Cases from CIUSSS-CHUS Hospital in Sherbrooke (N=12) were included in case counts but not in further analyses due to incomplete case reporting forms. ^3^Estimation of minimum incidence rate limited by under-reporting in Saskatchewan during first study period. For cohort 1, population estimates came from 2006 Canadian Census—Statistics Canada. For cohort 2, the reference population is the average population during those years of Canadian children aged 0**–**17 years.

**Supplementary Figure 1.** Histogram of bias adjusted incidence rate of non-type 1 diabetes per 100,000 children <18 years of age per year
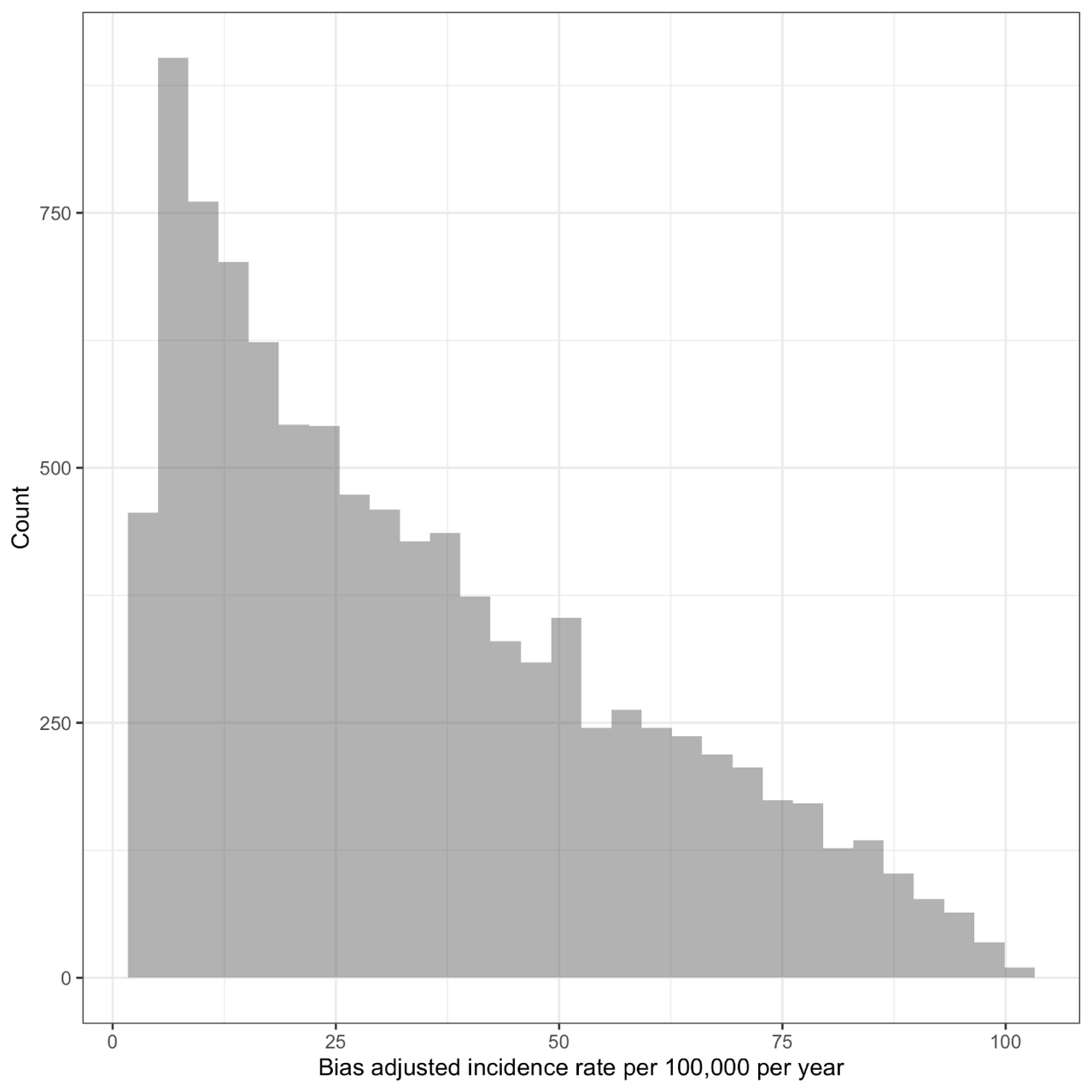

Supplement: Supplementary Materials — Table S1: case definition of non-Type 1 diabetes provided to reporting physicians. Table S2: inputs and distributions for the quantitative bias analyses (sensitivity analysis). Table S3: minimum incidence rates (per 100,000 children per year) for Type 2 diabetes, monogenic diabetes, and medication-induced diabetes for Cohorts 1 and 2 stratified by Canadian province. Figure S1: results of the sensitivity analysis in the form of a histogram showing the bias adjusted incidence rate per 100,000 per year of non-Type 1 diabetes. [file 5511049.f1.docx]
